# Bacteria-induced immunity: from homeostasis to immune subversion in periodontitis

**DOI:** 10.31744/einstein_journal/2026RW2369

**Published:** 2026-09-01

**Authors:** Vallentine Louzada Miranda, Mel de Araujo Teixeira, João Gabriel Silva Souza, Letícia Mello Bezinelli, Nídia Cristina Castro dos Santos

**Affiliations:** 1 Faculdade Israelita de Ciências da Saúde Albert Einstein Hospital Israelita Albert Einstein São Paulo SP Brazil Faculdade Israelita de Ciências da Saúde Albert Einstein, Hospital Israelita Albert Einstein, São Paulo, SP, Brazil.; 2 Divisão de Pesquisa Odontológica Universidade Guarulhos Guarulhos SP Brazil Divisão de Pesquisa Odontológica, Universidade Guarulhos, Guarulhos, SP, Brazil.; 3 Department of Oral Medicine, Infection, and Immunity Harvard School of Dental Medicine Harvard Medical School Boston MA United States Department of Oral Medicine, Infection, and Immunity, Harvard School of Dental Medicine, Harvard Medical School, Boston, MA, United States.

**Keywords:** Periodontitis, Innate immunity, Adaptive immunity, Dysbiosis, Inflammation, Host–microbe interactions

## Abstract

This review synthesizes current evidence on host immune responses to bacteria, their roles in the development and progression of periodontitis, and emerging immunomodulatory therapeutic strategies designed to restore periodontal homeostasis. Periodontitis develops through interactions between a dysbiotic bacterial community that disrupts host–microbe homeostasis and a chronic, maladaptive interaction with the host immune system. Key immune subversion microorganisms such as *Porphyromonas gingivalis* trigger inflammation while subverting immune surveillance by evading complement-mediated clearance and impairing neutrophil function. This dual behavior creates a biological paradox: bacteria induce inflammation to obtain nutrients while simultaneously suppressing effective antimicrobial activity, thereby perpetuating chronic, tissue-destructive immune activation. The osteoimmune response, particularly the Th17/IL-17 axis, is a central pathway linking microbial dysbiosis to persistent inflammation and alveolar bone resorption. These findings suggest that successful periodontal therapy must extend beyond mechanical biofilm removal by addressing underlying immune dysregulation. Potential adjunctive strategies for modulating this response include specialized pro-resolving mediators, complement inhibitors such as Cp40, and selected probiotics with immunoregulatory properties. Collectively, these approaches are intended to restore immune balance, enhance resolution of inflammation, and promote periodontal tissue repair. Current evidence demonstrates that periodontitis is not solely a microbial infection but also involves dysregulated host immunity. Clarifying how bacteria manipulate immune pathways may facilitate the development of therapies that complement bacterial control by targeting the inflammatory mechanisms underlying tissue destruction. Immunomodulatory interventions therefore represent promising candidates for promoting sustained periodontal stability and inflammatory resolution.

## INTRODUCTION

The bidirectional host-microbiota interaction is essential for systemic homeostasis.^(1,2)^ The innate immune system regulates this balance by recognizing pathogen-associated molecular patterns (PAMPs) and damage-associated molecular patterns (DAMPs).^(3,4)^The disruption of this ecosystem, known as dysbiosis, is a key etiological factor leading to the persistent activation of immune pathways and unresolved chronic inflammation.^(5,6)^

In this context, periodontitis is a destructive chronic inflammatory disease driven by a dysbiotic bacterial community that establishes a feedback loop with the immune response.^(7,8)^ Tissue damage, particularly alveolar bone resorption, is mediated by immune cells such as hyperactive Neutrophils and polarized Macrophages.^(9,10)^ In a crucial pathogenic mechanism, key periodontal pathogens, such as *Porphyromonas gingivalis*, actively subvert the host immune response – notably the inhibition of the complement system and crosstalk with TLR/C5aR receptors – thereby ensuring their survival and exacerbating tissue damage.^(11,12)^

Considering the central role of dysbiosis and unresolved inflammation, therapy must move beyond simple microbial elimination.^(13)^ Current immunomodulation strategies aim to reverse the chronic cycle. These include the use of specialized pro-resolving lipid mediators (SPMs) to reduce inflammation, promote efferocytosis, and facilitate the polarization of macrophages from the destructive M1 profile to the resolutive M2 profile.^(14-16)^ Additionally, the immunomodulatory properties of certain antibiotics, explored independently of their direct antimicrobial effects, represent an adjunctive therapeutic avenue. ^(17)^

In light of the foregoing, the present article aimed to conduct a detailed, integrative review of the literature on the cellular and molecular mechanisms of bacteria-induced immunity, and to apply this conceptual basis to critically discuss the pathogenesis of Periodontitis, with special emphasis on the immune subversion strategies employed by key pathogens to ensure their survival and induce tissue damage.

### BACTERIA-INDUCED IMMUNITY

#### General perspective

The interrelation between immunity and the microbiome extends beyond the simple balance between tolerance to commensal microorganisms and defense against pathogenic agents. The microbiota, in effect, integrates into the totality of the organism’s physiology, influencing numerous aspects of homeostasis through its impact on innate immunity. This immune system consequently acts as a regulator, adjusting to metabolic activity, dietary influences, exogenous substances, and mucosal infections. The collected information is processed at multiple physiological levels, allowing the host to adapt its activity to align with the microbial environment.^(1)^ In turn, the innate immune system plays a fundamental role in shaping the structure and composition of microbial communities, molding them into arrangements that are simultaneously tolerable and beneficial for the host’s metabolic needs. Therefore, the intrinsic and bidirectional relationship between the host and its microbiota proves essential for the maintenance of health (Figure 1).^(2)^

**Figure 1:**
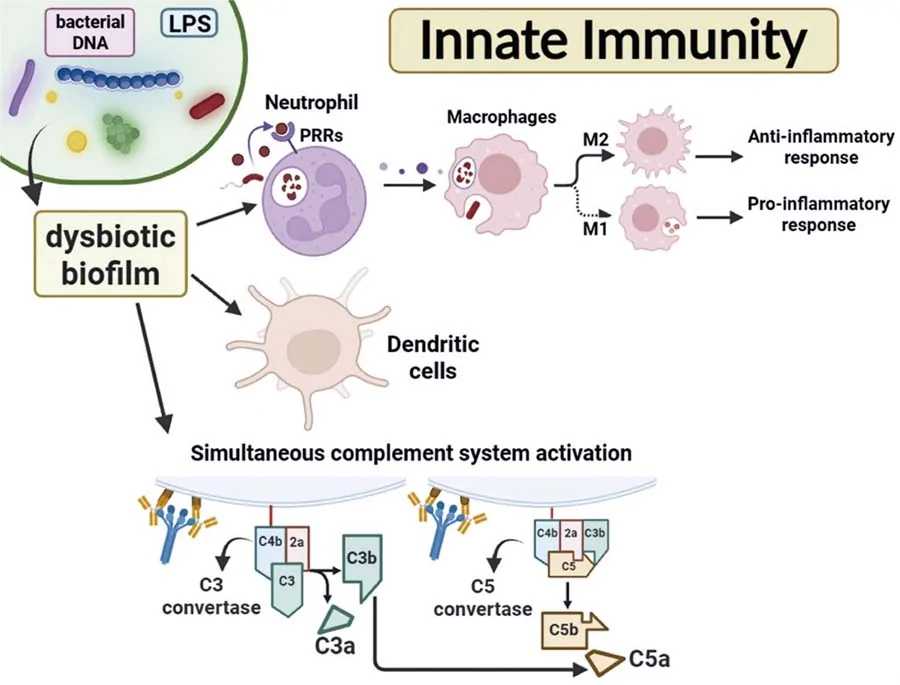
Interactions between dysbiotic biofilmes and humoral innate immune components

Created using the BioRender tool. Available from: https://www.biorender.com/

Host-microbiota interactions are crucial for the adequate maintenance of tissue homeostasis, and disruptions in these interactions are strongly correlated with the advent of various chronic pathologies. Among the key contributors to such diseases, PAMPs serve as persistent activators of immune responses, culminating in chronic and non-resolved inflammation.^(6)^ Additionally, anomalous microbial colonization during the maturation of the innate immune system can hinder the development of immunological tolerance, raising the risk of autoimmune and autoinflammatory diseases in later life stages.^(6,18)^ Furthermore, the microbiome substantially acts in the modulation of specific tissue immunity through mechanisms that influence not only local but also distant sites in the organism. As a result, imbalance of the microbiome, or dysbiosis, can trigger pathological changes in remote organs, producing symptoms in previously uncolonized tissues.^(2)^

#### Innate immune response to bacteria

The innate immune system employs a wide range of PRRs to identify conserved structural elements in pathogens, known as PAMPs, and endogenous danger signals that indicate host cell injury or stress, referred to as Damage-Associated Molecular Patterns (DAMPs).^(19)^ Pathogen-associated molecular patterns are specific and highly conserved molecular structures, shared by similar types of pathogens.^(4,20)^ These include various lipids, proteins, and nucleic acids, such as lipopolysaccharides (LPS), lipoteichoic acid (LTA), and bacterial DNA.^(3,21)^ Pathogen-associated molecular patterns are vital for pathogen survival and often possess unique molecular or subcellular characteristics, absent in host cells. This particularity allows innate immune cells, via PRRs, to identify PAMPs, differentiate non-self from self, and respond specifically to pathogens and their byproducts.^(3)^ Pathogen-associated molecular patterns encompass membrane-bound types that monitor the extracellular environment and endosomal compartments, such as Toll-like receptors (TLRs) and C-type lectin receptors (CLRs), as well as intracellular receptors, such as nucleotide-binding oligomerization domain (NOD)-like receptors (NLRs). This sophisticated receptor system allows the immune response to be finely modulated according to the type of pathogen (e.g., Gram-positive or Gram-negative bacteria, protozoa, viruses, and fungi) and the perceived threat level based on the pathogen’s location, whether inside the cytoplasm, in internal compartments, or outside the cell.^(22)^ PRRs are expressed by innate immune cells, including neutrophils, macrophages, dendritic cells (DCs), epithelial cells, and lymphocytes.

Neutrophils constitute the majority immune cell population in human blood, where they patrol and protect us from pathogens, and diseases like neutropenia show that they are indispensable for the control of bacterial and fungal infections.^(23)^ Neutrophils are fast, potent phagocytes endowed with a vast repertoire of cell-associated PRRs that, upon recognition of pathogens, apoptotic cells, or damaged senescent cells, promote neutrophil effector functions, such as the production of reactive oxygen species (ROS) and the secretion of antimicrobial peptides.^(24)^ Following the binding of opsonin receptors, such as Fcγ receptors, C-type lectins, or complement receptors, neutrophils engulf particles or microbes and release a variety of antimicrobial contents.^(23,25,26)^ At sites of infection, neutrophils are crucial in the defense against extracellular bacteria opsonized by complement and antibodies.^(27)^ Neutrophils can also release neutrophil extracellular traps (NETs), which is an extracellular fibrillar network formed by activated neutrophils and composed of nuclear components (e.g., DNA, histones) decorated by a set of proteins (e.g., myeloperoxidase (MPO), neutrophil elastase, lactoferrin, PTX3, MMP-9, and PGRP-S).^(27-29)^ Complementary to genomic DNA, mitochondrial DNA has been reported in NETs.^(30)^ In response to invading microorganisms, tissue-resident macrophages and mast cells activate the vascular endothelium through the secretion of cytokines, such as TNF-α and IL-1β, which promote neutrophil rolling, adhesion, and migration. Furthermore, neutrophils influence leukocyte recruitment through the release of pre-stored chemokines and cytokines, such as CXCL8, CCL2, and CCL20.

Neutrophils play a critical role in immune responses by releasing chemokines like CCL2 and CCL20, which facilitate the recruitment of interleukin-17-producing CD4+ T helper cells (Th17) to areas of infection or inflammation.^(31)^ Additionally, through the secretion of cytokines like B-lymphocyte stimulator and a proliferation-inducing ligand (APRIL), neutrophils support the survival, proliferation, and differentiation of B cells into antibody-producing plasma cells.^(32,33)^ Activated neutrophils under inflammatory conditions, such as those found in the synovial fluid of patients with rheumatoid arthritis, have also been shown to express the membrane-bound Receptor Activator of Nuclear Factor-κB Ligand (RANKL), an important osteoclastogenic factor,^(34,35)^ thus promoting osteoclastic bone resorption.^(36)^ Beyond pathogen elimination, recent discoveries reveal that neutrophils play a role in the resolution of inflammation after pathogen clearance.^(26)^ First, apoptotic forms of neutrophils trigger tissue repair in macrophages by promoting a regulatory IL-10 phenotype. Second, neutrophils switch from producing leukotriene B4 (LTB4) to lipoxin A4 (LXA4) in response to early eicosanoids, thereby reducing neutrophil infiltration. Neutrophils also contribute to the biosynthesis of omega-3-derived mediators (polyunsaturated fatty acids), namely resolvins and protectins, which further support tissue healing.^(37)^ Additionally, neutrophils constitute a heterogeneous population, exhibiting high degrees of functional plasticity with subsets adapted for a cytotoxic or regulatory character.^(38-43)^

Macrophages are present in all tissues, where they continuously monitor for signs of homeostasis disruption, while monocytes circulate in the blood, tracking intruders and rapidly moving to sites of infection.^(44)^ The type of mononuclear cells that interact with bacteria changes depending on the stage of infection – from initial colonization to established infection – and the nature of the disease, whether it is acute or chronic, localized or systemic. Macrophages have limited mobility but high phagocytic capacity, reflecting their primary function of protecting their resident tissues from infections. Upon detecting bacteria or danger signals, macrophages initiate autocrine feed-forward loops (such as TNF-α and IL-1β) and feedback loops (such as IL-10) as well as activate paracrine signaling to coordinate adaptive immunity and recruit blood-derived phagocytes.^(45)^

Macrophages exhibit significant heterogeneity and functional plasticity, adapting their activities in response to the surrounding microenvironment. Depending on local signals, macrophages can adopt M1 (classical) or M2 (alternative) activation states.^(14,45,46)^ M1 macrophages are activated by microbial components, such as LPS, and type 1 cytokines, such as IFN-γ. Compared to M2 macrophages, M1 cells are more proficient in microbial phagocytosis and express higher levels of pro-inflammatory cytokines, co-stimulatory molecules (such as CD86), and antimicrobial agents like ROS and nitric oxide. However, they exhibit a reduced capacity for efferocytosis, or the phagocytosis of apoptotic cells. These characteristics make M1 macrophages crucial for initial responses to infections and inflammation. Conversely, M2 macrophages, which can be activated by type 2 cytokines (such as IL-4 and IL-13), produce higher levels of immunoregulatory cytokines like IL-10 and TGF-β1. This profile confers roles on M2 macrophages in immune regulation, cell proliferation, and tissue repair.^(47-49)^

For certain pathogens, phagocytes are capable of achieving sterile eradication independently through their intrinsic effector functions. In contrast, other pathogens require phagocytes to receive support from additional immune cells. Parenchymal cells, for example, secrete antimicrobial peptides, chemokines, and cytokines to promote phagocyte chemotaxis, in addition to augmenting bacterial clearance. B cells produce antibodies to enhance pathogen recognition and phagocytosis, while T and NK cells activate additional antimicrobial mechanisms. Some highly persistent pathogens can survive even within activated phagocytes, necessitating additional tissue responses, such as granuloma or abscess formation, to effectively contain the infection.^(50,51)^

As a last innate immune resort, Natural Killer (NK) cells can be activated by the induction of NK cell activating ligand expression on infected cells and by the stimulation of DCs and macrophages to produce IL-12 and IL-15 (NK cell activating cytokines). NK cells produce IFN-γ, which, in turn, activates macrophages and enhances the elimination of phagocytosed bacteria.^(52,53)^ In this way, NK cells provide an early defense against these microorganisms before the development of adaptive immunity. Mice with severe combined immunodeficiency, lacking T and B cells, can temporarily control infection by the intracellular bacterium *Listeria monocytogenes* through NK cell-derived IFN-γ production. However, innate immunity generally fails to eradicate these infections, as complete clearance requires cell-mediated adaptive immunity.^(54)^

The Complement System is a cascading network of proteins and receptors that plays a key role in immunity and inflammation.^(55)^ Bacteria offer a surface that facilitates complement activation via the alternative pathway. Once PAMPs are detected on invading microorganisms, one or more complement initiation pathways are triggered, aiming to eliminate microbial intruders. Bacteria present mannose on their surface, which binds to mannose-binding lectin, or N-acetyl compounds that interact with ficolins, both initiating complement activation via the lectin pathway.^(56)^ Since microbes normally lack complement regulators, the response is rapidly amplified by the alternative pathway and results in opsonization, pro-inflammatory signaling, immune cell mobilization, and phagocytosis.^(55)^ Furthermore, the formation of the Membrane Attack Complex (MAC) through complement activation leads to bacterial lysis, with *Neisseria* species being particularly vulnerable due to their thin cell walls. Additionally, byproducts of complement activation promote inflammatory responses by recruiting and activating leukocytes.^(57)^

#### Adaptive immune response to bacteria

##### T Cells

T lymphocytes, the cells of cellular immunity, recognize antigens from cell-associated microorganisms, and different types of T cells assist phagocytes in destroying these microorganisms or eliminating infected cells. Their antigen receptors are distinct membrane molecules, but structurally related to antibodies. T cells possess a restricted specificity for antigens; they recognize peptides derived from foreign proteins that are bound to host proteins known as Major Histocompatibility Complex (MHC) Molecules which are expressed on the surfaces of other cells. T cells consist of functionally distinct populations, among which the most clearly defined are the T helper (Th) cells and the cytotoxic T lymphocytes (CTLs).^(58,59)^ The primary protective adaptive immune response against intracellular bacteria involves the recruitment and activation of phagocytes mediated by T cells. These cells provide defense against infections through two types of reactions: activation of phagocytes by CD4+ Th1 cells via the actions of the CD40 ligand and IFN-γ, leading to the elimination of microorganisms ingested by phagocytes in phagolysosomes, and CD8+ CTLs that eliminate microorganisms that escape phagocytic elimination mechanisms.^(60,61)^

Naïve CD4+ T cells, upon activation by antigen-presenting cells, such as DCs, can differentiate into specific T cell subsets – Th1, Th2, Th17, and regulatory T (Treg) cells – based on the cytokine environment present.^(62)^ Each subgroup is characterized by a unique cytokine profile, leading to distinct immune functions.^(63)^ Th1 cells play a key role in cell-mediated immunity against pathogens and similarly contribute to inflammatory and delayed-type hypersensitivity responses. Additionally, Th1 cells express CD40 ligand as well as secrete IFN-γ, and these two stimuli activate macrophages to produce various microbicidal substances, including nitric oxide, lysosomal enzymes, and ROS. These substances are primarily produced within phagolysosomes and, therefore, target microorganisms ingested inside the vesicles.^(64)^ Th2 cells, which secrete IL-4, IL-5, and IL-13, are involved in humoral immunity, promoting IgE production and mast cell activation, and play roles in allergic responses. Th17 cells release IL-17A, IL-17F, IL-21, and IL-22, which help recruit neutrophils and mount immunity against extracellular bacteria and fungi, but are also associated with autoimmune and inflammatory diseases, as well as bone resorption.^(64)^ Treg cells, characterized by the production of IL-10 alongside TGF-β, are crucial for immune regulation, maintaining self-tolerance and suppressing excessive inflammation through the negative regulation of Th1, Th2, and Th17 cell activity.^(7,55,65)^

Th17 cells are found at barrier sites, such as the gastrointestinal tract (GIT) and the skin, where they play crucial roles in immune protection and barrier integrity, functions closely linked to the cytokine IL-17A. Produced by Th17 cells, IL-17A supports epithelial defenses, strengthens barrier integrity, and recruits neutrophils in response to injury or infection, facilitating antibacterial and antifungal defenses at these surfaces.^(66,67)^ Specific bacteria in the GIT and skin are known to stimulate the differentiation of Th17 cells; for example, segmented filamentous bacteria (SFB) in the gut and Staphylococcus epidermidis on the skin were among the first commensals identified to induce Th17 cell responses in healthy contexts.^(68,69)^ The antigenic specificity of Th17 cells for commensal microbes has also been demonstrated in the gut, where they selectively recognize commensal antigens.^(70)^ In disease contexts, commensal-induced Th17 responses have been implicated; SFB stimulates Th17 activity not only during health but also in inflammation, and *Eggerthella lenta*, an Actinobacterium associated with inflammatory bowel disease, has been shown to promote Th17 induction in both mice and humans.^(71)^ Furthermore, Bifidobacterium adolescent is was found to induce Th17 in an antigen-specific manner, exacerbating the severity of arthritis in murine models.^(71)^

##### B Cells

B lymphocytes are primarily known for their contribution to immunity through antibody production, antigen presentation, and cytokine production. Humoral immunity is mediated by B cells and plasma cells, serving as a defense mechanism against extracellular microorganisms. Antibodies secreted by B cells prevent infections and eliminate pathogens through antigen recognition, induction of phagocytosis, and complement activation.^(58,59)^ However, a subpopulation of B cells, namely regulatory B cells (Bregs), has a suppressive role in bacteria-induced immunity through various mechanisms: induction of Tregs via the production of IL-10, IL-35, and TGF-β;^(72)^ inhibition of T cell differentiation into Th1 and Th17 by suppressing pro-inflammatory cytokine production by DCs;^(72)^ and reduction of phagocytosis and antigen presentation by macrophages.^(73)^

B cells have an important role in the pathogenesis of tuberculosis. When naïve B cells identify Mycobacterium tuberculosis, they differentiate into plasma cells, secreting antibodies that lead to pathogen opsonization, induce antibody-dependent cellular cytotoxicity, and neutralize M. tuberculosis antigens.^(74)^ Subsequently, the suppressive activity of Bregs may play a fundamental role in the pathogenesis of tuberculosis infection, with high levels of Bregs hindering an effective anti-tuberculosis immune response.^(75)^

## BACTERIA-ASSOCIATED IMMUNE RESPONSE IN PERIODONTITIS

### General perspective

Periodontitis constitutes a chronic inflammatory disease that affects the tissues supporting the teeth, collectively known as the periodontium.^(76,77)^ This chronic inflammatory condition is associated with a biofilm composed of communities,^(77)^ that organize into complex spatial structures.^(8,78)^

Microbial communities can be defined by their composition, function, and spatial structure.^(79-82)^ Such communities are highly dynamic at spatial, temporal, and phylogenetic levels, which, in turn, modulates phenotypic diversity and functional activities.^(83-85)^ Changes in host immune competence or diet can influence the composition of microbial communities and shift the metatranscriptional profile, often increasing the production of virulence factors. As the microbial community evolves, microbial metabolism and the byproducts of the host immune response can alter the local environment, promoting the expansion or over-representation of microorganisms linked to a dysbiotic state.^(8)^

### Dysbiosis and inflammation

Periodontitis is driven by mutually reinforcing interactions between a polymicrobial dysbiotic community and an amplified host inflammatory response.^(8,78)^ Inflammation plays a fundamental role in driving the microbial population shift characteristic of periodontal disease progression. The inflammatory environment creates selective pressures on the microbial community.^(8,86)^ Inflammation can promote the overgrowth of periodontitis-associated microorganisms through tissue destruction that releases nutrients, such as degraded collagen and heme-containing compounds, which are sources of amino acids and iron.^(12)^ These nutrients can be transported via the Gingival Crevicular Fluid (GCF) into the gingival sulcus/pocket to foster the growth of subgingival proteolytic and asaccharolytic bacteria with iron-acquiring capacity.

The question of whether the dysbiotic microbiome directly causes the inflammatory pathology or is simply a byproduct of a nutritionally favorable inflammatory environment has only recently been explored through controlled microbiome transfer experiments. In the context of periodontitis, there appears to be a reciprocal cause-and-effect relationship between dysbiosis and inflammation. Inflammation promotes the selective growth of dysbitic communities, while dysbiosis, in turn, exacerbates inflammation.^(12,13,87-91)^ Thus, it is suggested that neither destructive inflammation nor dysbiosis can fully manifest without interactions between these two processes. In summary, inflammation serves as a reciprocal ecological driver of dysbiosis, and the interaction between inflammation and dysbiosis appears to create a self-sustaining feedforward-loop that contributes to the progression of periodontitis.^(86,92)^

It has been demonstrated that the dysbiotic microbiome induced by *P. gingivalis* can be stably transferred to germ-free mice, leading to the development of periodontitis in the recipient mice.^(93)^ The study further demonstrated that the *P. gingivalis*-induced dysbiotic microbiome can be vertically transmitted from parents to their offspring, resulting in considerable bone loss in these offspring compared to those derived from periodontally healthy mice. Although antibiotic treatment successfully reversed dysbiosis in the periodontal microbiome of *P. gingivalis-*challenged mice – impacting both microbial load and community structure – the dysbiotic condition rapidly reappeared once treatment was discontinued.^(93)^

The central function of inflammation in the development of dysbiosis presents a biological paradox: dysbiotic communities require inflammation to obtain nutrients, yet simultaneously need to negatively regulate the host immune response to protect themselves.^(8,78)^

Periodontal bacteria can manipulate their interactions with host immune responses – such as those involving neutrophils and complement – to increase their biological fitness (survival).^(12)^

### Bacterial subversion of the immune response

Bacteria associated with periodontitis, including *P. gingivalis, Tannerella forsythia,* and *Prevotella intermedia*, can manipulate the complement system. These bacteria protect themselves and adjacent bacteria from opsonization and elimination by human complement by obstructing complement activation. This evasion can occur through the degradation of C3 or by targeting crucial upstream components, such as mannose-binding lectin.^(94-96)^ Bacterial proteases produced by *P. gingivalis, T. forsythia, and P. intermedia* act synergistically to inhibit complement activity, which can maximize protection of nearby species susceptible to complement.^(94-96)^ Furthermore, P. intermedia and Treponema denticola can bypass elimination by human complement by capturing complement factor H, a physiological inhibitor of the complement pathway.^(97,98)^

It is acknowledged that key immune subversion microorganisms utilize a range of strategies to protect contiguous bacteria and enhance the virulence of the entire microbial community. *P. gingivalis* has been shown to degrade and neutralize antimicrobial peptides, offering protection to nearby bacteria in vivo.^(99)^ In a related context, *T. denticola* impairs the production of human β-defensins by gingival epithelial cells in response to *F. nucleatum*, thereby promoting the colonization of periodontitis-associated bacteria.^(100)^ Mechanistically, *T. denticola* inhibits the fusion of internalized *F. nucleatum* with lysosomes and reduces intracellular ROS production, which in turn suppresses TLR responses that are crucial for regulating human β-defensin expression (Figure 2).^(100)^

**Figure 2:**
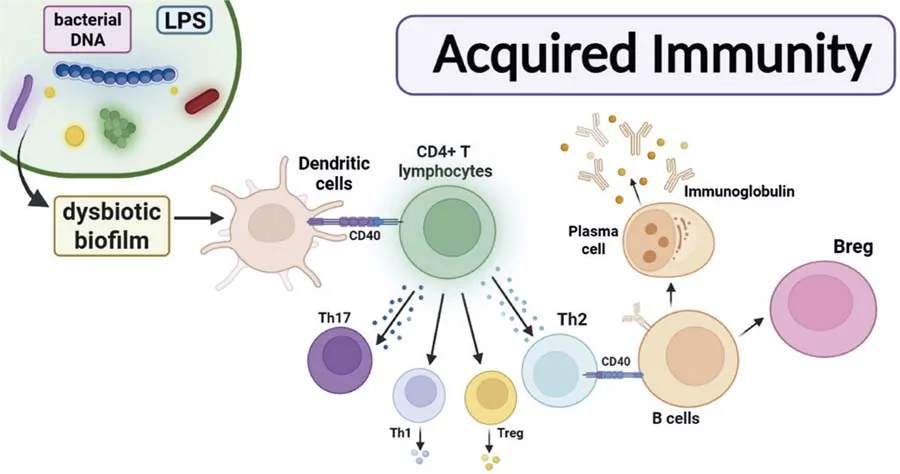
The acquired immune response cascade: from antigen presentation to immunoglobulin production

Created using the BioRender tool. Available from: https://www.biorender.com/

### Innate immunity in periodontitis

Neutrophils are the predominant leukocytes recruited to the subgingival sulcus or periodontal pockets.^(101-103)^ Individuals with congenital deficiencies in neutrophil number or recruitment exhibit severe periodontitis, indicating that neutrophils are essential for maintaining periodontal tissue homeostasis.^(5,104,105)^ However, hyperactive, excessive, or dysregulated neutrophils can lead to tissue damage through the release of inflammatory and toxic substances, as well as tissue-degrading enzymes.^(10,106)^

Periodontal health is highly dependent on proper neutrophil function, with hypo- and hyper-responsiveness linked to dysregulated inflammatory responses and bone loss.^(104)^ For example, LAD-1 deficiency, a rare condition that affects neutrophil extravasation, and Papillon-Lefèvre syndrome, which affects neutrophil functionality, can lead to a severe and rapidly progressive form of periodontitis.^(9)^ When neutrophils are unable to infiltrate the gingiva, there is an excessive production of IL-23, IL-17, and G-CSF in the periodontium, driven primarily by macrophages. This cascade results in increased inflammation and osteoclastogenesis.^(105)^ Conversely, their local abundance positively correlates with the severity of chronic periodontitis.^(107)^ The chronic influx of excessive neutrophils into diseased periodontal pockets likely stems from their inability to properly control microbial challenges, although they are viable and capable of eliciting immune responses.^(108)^ This indicates that microorganisms can evade neutrophil-mediated elimination while simultaneously promoting inflammation, thus contributing to dysbiosis. Additionally, neutrophils with a hyper-inflammatory phenotype overproduce ROS and pro-inflammatory cytokines, such as IL-1β, IL-6, IL-8, and TNF-α. This hyperactivity, combined with defects in functions such as phagocytosis and chemotaxis, can be associated with further tissue damage and an increased risk of comorbidity with other inflammatory diseases.^(109,110)^

*P. gingivalis* employs various virulence factors to disrupt neutrophil responses. *P. gingivalis* can inhibit opsonization and phagocytosis while enhancing neutrophil recruitment and respiratory burst, thereby exacerbating neutrophil-associated inflammation and tissue damage.^(111)^ Furthermore, LPS-induced TLR2 activation and crosstalk with C5aR inhibit the MyD88 pathway while activating the Mal-PI3K pathway, leading to the suppression of antimicrobial responses and PI3K-mediated phagocytosis, analogously promoting Mal-dependent inflammation.^(11)^

Recent evidence suggests that *F. nucleatum* elicits responses in human neutrophils that are subspecies-specific.^(112)^ By stimulating human neutrophils with different *F. nucleatum* subspecies [*F. nucleatum ssp. animalis* (*Fna*), *F. nucleatum ssp. fusiforme* (*Fnf*), *F. nucleatum ssp. nucleatum* (*Fnn*), *F. nucleatum ssp. polymorphum* (*Fnp*), *F. nucleatum ssp. vicentii* (*Fnv*)], it was observed that the immunogenicity of *F. nucleatum* in human neutrophils is highly subspecies-specific in vitro regarding ROS release and cytokine production. Subspecies *Fna* and *Fnp* stimulated high superoxide release and low cytokine release, while *Fnv* conversely stimulated low superoxide release and high cytokine release.^(113)^ These findings suggest that the immune response induced by *Fn* should be studied considering individual subspecies, which may contribute to unraveling physiological and genetic similarities and distinctions between these bacteria.^(114)^

Although macrophages are present in low numbers within periodontal tissues,^(115)^ they play a crucial role in the pathogenesis of periodontitis by initiating and resolving inflammation, assisting in tissue repair, activating lymphocyte-mediated adaptive immunity, and mediating alveolar bone resorption and apposition.^(116)^ During periods of inflammation, tissue-resident macrophages expand and circulating monocytes are recruited to differentiate into macrophage-like cells.^(117)^

Macrophages are capable of phagocytosing periodontal bacteria that infiltrate the gingival connective tissue. However, *P. gingivalis* can induce cAMP-dependent activation of protein kinase A, which inhibits inducible nitric oxide synthase expression, thereby suppressing nitric oxide-dependent intracellular clearance in macrophages.^(118)^ Additionally, *P. gingivalis* obstructs the endocytosis of *F. nucleatum* by human and murine macrophages, which is critical for NLRP3 inflammasome activation in response to this bacterium.^(118)^ This mechanism can enhance the survival of the periodontal microbial community, as inflammasome activation triggers pyroptosis – a pro-inflammatory form of lytic cell death that serves to protect the host from infection.^(12,119)^

Both M1 and M2 macrophages are elevated in periodontitis compared to healthy controls; however, the M1/M2 ratio is skewed towards M1 in periodontitis, correlating with increased expression of M1-associated molecules such as IL-1β, IL-6, and matrix metalloproteinase (MMP)-9.^(120)^ Experimental periodontitis influences circulating monocytes/macrophages, which often exhibit an M1 phenotype characterized by exacerbated expression of TNF-α and IL-6.^(121)^ A temporal analysis of the inflammatory response in the healing of osteolytic periodontal lesions demonstrated changes in macrophage activation from an inflammatory phenotype (marked by CD80 and TNF-α expression) to a resolutive phenotype (indicated by CD206 expression), correlating with the extent of bone loss.^(122)^

### Th17 and osteoimmunology

The term “osteoimmunology” was introduced over two decades ago to describe the interdisciplinary field focused on the interactions and regulatory mechanisms between bone cells and the immune system.^(123)^ Th17 cells and their associated signaling pathways exert a significant role in immune-bone interactions that are relevant to the pathogenesis of inflammatory diseases with bone loss, such as rheumatoid arthritis, ankylosing spondylitis, osteoarthritis, and periodontitis.^(124)^ Specifically, the Th17/IL-17 axis has been implicated in the pathogenesis of periodontitis, similarly to other inflammatory bone disorders. Multiple studies have consistently demonstrated the upregulation of Th17-related cytokines, including IL-17A, IL-17F, IL-22, IL-23, and IL-21, in tissue lesions of patients with periodontal disease under the regulation of the transcription factor RORgamma and the critical involvement of STAT3.^(125)^ Notably, cytokine levels such as IL-17A, IL-23, and IL-21 in periodontal mucosal tissues have been correlated with the extent of bone destruction.^(126-128)^ Additionally, the expansion of Th17 cells has been documented in both common and rare genetic forms of periodontitis.^(105)^

A distinctive feature of periodontitis, in contrast to other inflammatory bone diseases such as rheumatoid arthritis and osteoarthritis, is its initiation by a dysbiotic microbiome.^(129,130)^ In cases of autoimmune inflammatory bone loss, Th17 cells are typically activated by specific autoantigens. However, in periodontitis models, the expansion of Th17 cells occurs in oral mucosal gingival tissues in response to the presence of a dysbiotic microbiome.^(130,131)^ In experimental models of periodontal disease, Th17 cells are known to accumulate after disease induction^(130-132)^ and are recognized for their pathogenic role as key drivers of inflammation and bone destruction in these models. Humans deficient in Th17 differentiation, resulting from a loss-of-function mutation in the *STAT3* gene, exhibit significantly reduced levels of Th17 cells in both circulation and gingival tissues. This deficiency is associated with lower gingival inflammation and less periodontal bone loss compared to the general population.^(131)^

Th17 cells are the most osteoclastogenic T cell subtype, directly inducing RANKL expression in resident cells through the production of IL-17.^(133,134)^ In healthy individuals, Th17 cells naturally accumulate in the gingival mucosa with age, contributing to barrier defense. This accumulation is driven by mechanical stimulation, such as mastication, which induces IL-6 production in epithelial cells and occurs independently of commensal bacteria.^(133)^ Conversely, the expansion of Th17 cells during periodontitis is contingent upon microbial dysbiosis and requires the production of both IL-6 and IL-23.^(131)^ These cells consist predominantly of resident memory Th17 cells, which are capable of rapid responses and serve as the main producers of IL-17.^(133)^ A study in mice demonstrated that IL-17- producing Th17 cells play a significant role in periodontitis-associated bone damage, emphasizing that IL-17 production is crucial for host defense against oral bacterial invasion.^(130)^

Although the amplification of Th17 cells during disease has been shown to be microbe-dependent in experimental periodontitis models, specific microbial constituents implicated in the induction of disease-associated Th17 responses have not yet been identified in either mice or humans. Additionally, it remains uncertain whether Th17 cells in periodontitis exhibit antigenic specificity toward specific disease-associated pathobionts.^(67)^

### B Cells

B cells are virtually absent in normal gingival mucosa,^(115,135)^ however, they dramatically increase as the disease progresses, becoming a distinctive feature of the established periodontal lesion.^(7,136)^ During periodontitis, stromal cells and immune cells express different cytokines and chemokines, such as IL-4, IL-6, IL-5, CXCL13, and APRIL, which induce B cell migration and sustain their survival in the periodontium.^(137,138)^ B cells/plasma cells are well-known for their humoral immunity. The B cell-mediated, IgG-dominated immune response may contribute to the pathogenesis of periodontitis.^(137,139)^ The majority of infiltrating B cells present during periodontitis produce RANKL, suggesting they can directly induce osteoclastogenesis.^(140)^ Indeed, the induction of experimental periodontitis in B cell-deficient mice demonstrated significantly reduced bone loss.^(133,136)^

## CLINICAL IMPLICATIONS FOR PERIODONTAL THERAPY

Periodontitis is a chronic inflammatory disease associated with a dysbiotic biofilm.^(13)^ The standard treatment for periodontitis primarily involves promoting behavioral changes to improve patient-performed supragingival biofilm control, professional mechanical removal of the biofilm, risk factor management, as well as subgingival instrumentation.^(141)^ Although non-surgical treatment aims to combat the etiological biofilm, patients with severe tissue destruction and hyper-inflammatory responses may not consistently achieve optimal clinical outcomes.^(142-144)^ Thus, immune modulators have been proposed as adjuvant therapies (Figure 3).

**Figure 3:**
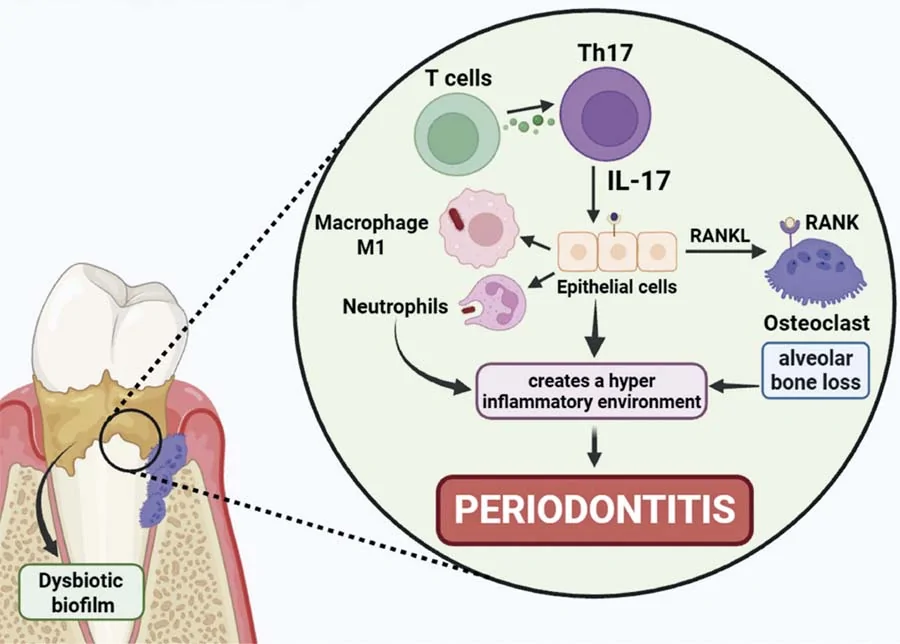
Mechanisms of osteoclastogenesis and bone destruction in the inflammatory environment of periodontitis

Created using the BioRender tool. Available from: https://www.biorender.com/

Current evidence suggests that high-dose omega-3 supplementation can improve clinical outcomes, including clinical attachment level (CAL) gain and probing pocket depth (PPD) reduction.^(145-154)^Additionally, omega-3 supplementation may help reduce pro-inflammatory cytokines and RANKL in saliva, gingival crevicular fluid, and serum at 3 and 6 months after periodontal treatment, compared to placebo groups.^(155)^ However, the adjuvant benefits of omega-3 in periodontal therapy require confirmation through large-scale, long-term clinical trials.

Probiotics have also been investigated as host-modulating agents in multiple clinical trials, with findings indicating that *Lactobacillus reuteri*^(156-161)^ and *Bifidobacterium animalis subsp. lactis*^(162-164)^ can achieve greater reductions in PPD and CAL gain when compared to control groups. However, meta-analyses comparing the efficacy of probiotics in periodontal treatment have yielded contrasting conclusions, with one reporting better clinical outcomes favoring probiotics,^(165)^ while another found no significant benefit in periodontal parameters.^(166)^ The term ‘probiotics’ encompasses a variety of therapeutic approaches involving different bacterial species and strains, formulations, and protocols. Therefore, additional clinical trials with standardized probiotic regimens are recommended to clarify their adjuvant value.

Regarding bisphosphonates, local application of 1% alendronate gel (10mg/mL) has shown potential in improving CAL and reducing PPD in periodontitis patients, including both normoglycemic individuals^(167)^ and those with type 2 diabetes.^(168)^ Nevertheless, due to the potential adverse effects associated with bisphosphonates, the clinical advantages of using alendronate in periodontitis must be carefully evaluated in further trials.

Current evidence suggests that the local application of statins can benefit the treatment of periodontal pockets. Two systematic reviews reported significant reductions in PPD and CAL gain with the topical application of simvastatin, atorvastatin, and rosuvastatin at 3 and 6 months following periodontal therapy.^(166,169)^ However, much of the data supporting the application of statins in periodontal pockets stems from a single research group and has not yet been independently replicated. Furthermore, systemic administration of statins has not demonstrated periodontal benefits in human trials, and continuous systemic use of statins specifically for periodontal therapy is not recommended.^(169,170)^

Multiple preclinical studies^(15,131,171-174)^ and a Phase 1 clinical trial^(175)^ show that SPMs can effectively promote immunomodulation in periodontitis. Specialized pro-resolving lipid mediators have been shown to reduce inflammation, modulate the secretion of pro- and anti-inflammatory mediators, enhance M1 to M2 macrophage polarization, downregulate MHC class II expression, and inhibit CD4+ T cell activation. Additionally, SPMs facilitate alveolar and craniofacial bone regeneration in inflammatory environments, both with and without the presence of xenograft or synthetic grafts.^(176)^ In murine models, resolvin E1 reversed periodontitis progression and modulated the subgingival microbiota, highlighting the impact of localized immune modulation on microbiota composition.^(16)^ These findings highlight SPMs as promising therapeutic interventions for periodontitis treatment and the promotion of alveolar bone preservation.

Complement inhibition has also demonstrated beneficial effects in periodontitis treatment in preclinical studies^(177,178)^ and a Phase IIa clinical trial.^(175)^ The C3 inhibitor Cp40 (AMY-101), administered locally, showed potent anti-inflammatory effects. In non-human primates, Cp40 effectively reduced gingival bleeding, PPD, and tooth mobility,^(177)^ as well as the activation of proteins in the alternative complement pathway in gingival crevicular fluid (GCF).^(178)^ In a human trial, Cp40 demonstrated a reduction in gingival inflammation.^(175)^ These findings suggest that complement inhibitors, such as Cp40, may offer a novel approach to the management of periodontal inflammation.

*In vitro* and *in vivo* studies have shown that antibiotics can modulate immune responses in various inflammatory conditions, independent of infectious origins.^(174)^ *In vitro*, antibiotics have been shown to modulate immune responses in myeloid and epithelial cells by decreasing the production of pro-inflammatory cytokines and free radicals, reducing NETs release, and diminishing nitric oxide synthesis in lymphocytes.^(174,179-183)^Complementarily, the immunomodulatory properties of antibiotics were investigated in animal and human models of infection-associated inflammation or bacterial stimulation, such as periodontitis, where antibiotic treatment led to reduced levels of inflammatory markers.^(181)^ In response to LPS stimulation, antibiotics led myeloid cells, epithelial cells, and periodontal ligament cells to modulate cytokine production, enhance M2 macrophage polarization, and reduce TLR expression in DCs.^(184-186)^

Collectively, current evidence suggests that dietary omega-3 supplementation and specific probiotics have beneficial effects on periodontitis treatment. Although bisphosphonates present positive results, data supporting their clinical use remain limited. Novel therapeutic candidates, namely SPMs and Cp40, represent promising approaches for periodontitis treatment. Antibiotics exhibit immunomodulatory potential in periodontitis, mitigating inflammation and reducing tissue-destructive immune responses, in addition to their antimicrobial effects.

## CONCLUSION

The dysfunctional bacterium-immune system interaction, characterized by dysbiosis and subversion of the host immune response, is the primary mechanism driving the pathogenesis of Periodontitis. Effective therapy must aim beyond microbial elimination, focusing on resolutive immunomodulation (e.g., via specialized pro-resolving lipid mediators) to reverse the chronic inflammatory cycle.
